# Effects of oxytocin versus promestriene on genitourinary syndrome: a pilot, prospective, randomized, double-blind study

**DOI:** 10.1016/j.clinsp.2022.100116

**Published:** 2022-10-01

**Authors:** Liani Patricia Andrade Santos, Claudio Emílio Bonduki, Rita de Cássia de Maio Dardes, Thais Heinke, Marisa Teresinha Patriarca

**Affiliations:** a Departamento de Ginecologia, Universidade Federal de São Paulo (UNIFESP), São Paulo, SP, Brasil; b Departamento de Patologia, Universidade Federal de São Paulo (UNIFESP), São Paulo, SP, Brasil

**Keywords:** Genitourinary Syndrome, Promestriene, Oxytocin, Vulvovaginal Atrophy, Menopause

## Abstract

**Objectives:**

In this pilot, prospective, randomized, double-blind study, the authors compared the efficacy of oxytocin with promestriene in improving vaginal atrophy of Genitourinary Syndrome of Menopause (GSM).

**Methods:**

A total of 51 postmenopausal women with symptoms of GSM were evaluated. They were randomized into two groups: oxytocin (25 patients) and promestriene (26 patients) and were evaluated before and after 90 days of treatment; the evaluation was based on the domains of the Female Sexual Function Index (FSFI) (lubrication, satisfaction, and pain during sexual intercourse), clinical visual examination, and vaginal wall thickness.

**Results:**

After the use of the medications, both groups showed significant improvement in the three evaluated FSFI domains (p < 0.05) and there was no significant difference between the groups (p > 0.05). On clinical examination, the medications improved all the evaluated parameters but without statistical significance (p > 0.05). The evaluation of the thickness of the vaginal epithelium showed that both treatments led to increase in the vaginal epithelium (p < 0.05); however, the efficacy of promestriene was higher than that of oxytocin (p < 0.05).

**Conclusions:**

Both medications were effective, however, studies with larger samples and longer follow-ups are needed to confirm the clinical applicability.

## Highlights

• Prospective, double-blind, randomized pilot study.
• Compares efficacy of oxytocin and promestriene in Genitourinary Menopause Syndrome.
• The two medications improved the three domains of the FSFI scales scores evaluated.
• Both medications showed an increase in the thickness of the vaginal epithelium (p < 0.05).
• Oxytocin and promestriene were both effective in Genitourinary Menopause Syndrome.

## Introduction

The term Genitourinary Syndrome of menopause (GSM) was first defined by the North American Menopause Society in 2013^1^ and officially confirmed by the International Society for the Study of Women's Sexual Health in 2014.^2^ The new nomenclature defines a chronic and progressive condition characterized by signs and symptoms that occur in the genitourinary tract as a result of postmenopausal hypoestrogenism.^2^, ^3^, ^4^, ^5^

The incidence of GSM increases with years after menopause and affects between 40% and 57% of women in this stage of life.^2^^,^^3^^,^^6^^,^^7^

The clinical manifestations of GSM resulting from estrogen deficiency include symptoms related to the genitalia, urinary tract, and sexual function.^3^^,^^8^^,^^9^^,^^10^

The main objective of GSM therapy is the improvement of symptoms related to vaginal atrophy imposed by estrogen deficiency. The treatment options are varied and include local and/or systemic hormonal therapy, lifestyle changes, and nonhormonal treatments.^4^^,^^8^^,^^11^^,^^12^ When symptoms of GSM manifest in isolation, the treatment of choice is estrogen therapy via the vaginal route in low doses.^1^^,^^13^

There is evidence to show that promestriene, a diether of estradiol, is effective for the treatment of GSM with negligible systemic absorption.^14^ Due to low plasma concentration, it acts on the vaginal mucosa and does not stimulate the endometrium.^15^^,^^16^

In the last decade, some studies have indicated the use of oxytocin via the vaginal route as an alternative and promising treatment for the relief of vasomotor symptoms related to GSM.^17^, ^18^, ^19^, ^20^, ^21^ Oxytocin receptors are found in the vaginal epithelium, and oxytocin stimulates the proliferation of epithelial cells, in addition to stimulating blood flow and local metabolism.^22^ Oxytocin has been shown to increase vaginal mucosal blood flow and nutrient transport as well as to stimulate the secretion of various growth factors and induce mitosis in various types of cells.^23^

The effect of oxytocin in the vagina is local, and the increase in oxytocin production and release does not seem to interfere with the function of estrogen or its receptors.^17^^,^^24^ Conversely, in experimental studies, the estrogen stimulus was able to sensitize the oxytocin gene promoter with a direct effect on oxytocin transcription, production, and release.^24^ Thus, some researchers have indicated the vaginal use of oxytocin for the treatment of GSM in postmenopausal women for whom estrogen therapy is contraindicated.^25^

A recent meta-analysis showed evidence that oxytocin is beneficial and safe in the treatment of vaginal atrophy, without significant endometrial stimulation and/or increased blood levels of estradiol.^26^

Vaginal oxytocin has been shown to be promising for the treatment of clinical manifestations of GSM and may be considered an alternative for patients with contraindications to estrogen therapy. The lack of dose standardization and the lack of prospective studies on the subject encouraged us to investigate the effectiveness of oxytocin in alleviating symptoms related to urogenital atrophy, as well as comparing it to the well-established effects of promestriene.

## Methods

This pilot, prospective, randomized, double-blind study was conducted at the Climacteric Outpatient Clinic of Universidade Federal de São Paulo ‒ Escola Paulista de Medicina (UNIFESP-EPM) together with the Center for Women's Integrated Health Care (Aracaju/SE) between September 2018 and January 2020. The study was approved by the Research Ethics Committee (CEP) of UNIFESP and registered in the Brazilian Registry of Clinical Trials (REBEC) under UTN number: u1111-1239-1765.

In the preselection phase, women who presented signs and symptoms related to genitourinary atrophy after menopause were evaluated (vaginal dryness, pain and/or discomfort during sexual intercourse, vulvar and/or vaginal itching, paleness of the vaginal mucosa, reduction of rugae, and friability) by anamnesis and by physical, general, and gynecological examination. In addition, complementary exams considered routine in the specialty were requested.

The authors used the FSFI scale, adapted to the Portuguese language,^27^^,^^28^ to assess and quantify the women's subjective complaints related to sexual intercourse. The FSFI scale consists of 19 questions grouped into 6 domains: desire, arousal, vaginal lubrication, orgasm, sexual satisfaction, and pain or discomfort. The score for each domain is multiplied by a specific factor (0.6 for desire, 0.3 for arousal and lubrication, and 0.4 for the remaining domains) and the result corresponds to a weighted score. The final FSFI score is obtained by adding the weighted scores of each domain.^27^

In this study, the applied questionnaire had 10 questions from the FSFI scale and quantified 3 of the 6 recommended domains (lubrication, four questions; satisfaction, three questions; pain or discomfort, three questions). The domains were quantified, and the weighted score for each domain was obtained. These domains were chosen considering that the objective was to assess and quantify the symptoms directly related to vaginal atrophy; therefore, the authors considered that desire, arousal, and orgasm did not meet this criterion. Using the FSFI domains was the approach used to quantify qualitative variables.

**Table:** 

**Domain**	**Questions**	**Value of answer**	**Factor**	**Minimum score**	**Maximum score**
Lubrication	1, 2, 3, 4	0 to 5	0.3	0	6
Satisfaction	5, 6, 7	0 to 5	0.4	0	6
Pain	8, 9, 10	0 to 5	0.4	0	6

*Adapted (3 domains: 10 questions) from ROSEN, C., et al. (28). The Female Sexual Function Index (FSFI): a multidimensional self-report instrument for the assessment of female sexual function. Journal of sex & marital therapy, v. 26, n. 2, p. 191-208, 2000.^28^

The following inclusion criteria were used: Women with > 2 years postmenopause and a level of follicle stimulating hormone of > 40 mU/mL; Women with signs and symptoms related to GSM; Women who had not used any hormonal medication (systemic and/or topical) for the treatment of climacteric symptoms in the last 12 months; Women who were not using vaginal lubricants or hydrants for 6 months; Women with cervicovaginal cytology of an atrophic pattern performed in the last 12 months must be recorded in medical records.

Women who had the following were excluded from the study: Diseases known or suspected to be estrogen-dependent, including neoplastic and nonneoplastic diseases; A history of vulvovaginal dermatoses such as lichen sclerosis, lichen planus, and condylomas or a history of pelvic irradiation; Genital infections, including herpes simplex, bacterial vaginosis, and yeast; Gynecological routine exams not updated in the last 12 months or showing alterations (including mammography, cervical-vaginal cytology test, and transvaginal ultrasound to assess endometrial echo).

Thus, 51 postmenopausal women were selected (convenience sample because this was a pilot study). All participants received oral and written instructions in the prescription about the treatment and signed an informed consent form in accordance with the norms of the Research Ethics Committee of UNIFESP-EPM, which approved the study.

The women were randomized into two distinct groups, with women being given an equal chance of being assigned to either group. The randomization code was only broken when all assessments had been performed and the results were entered into the database.

The oxytocin group (25 cases) received vaginal oxytocin gel 1g (300 IU), and the promestriene group (26 cases) received vaginal promestriene 1g (10 mg/g). The substances were provided by the researcher in identical packaging and with a standardized applicator for both groups so that women received the same amount of medication. The women were instructed to apply the product vaginally at night (at bedtime) and after bathing. The initial single daily concentration was 300 IU/g of oxytocin or 10 mg/g of promestriene for 15 consecutive nights, and the same dose was used for maintenance, administered three times a week (on nonconsecutive days) for the remaining 75 days, totaling 3 months of treatment. Patient assessment was performed at three visits during the study.

First visit: Anamnesis, gynecological examination, and vaginal biopsy were performed. Women responded to a questionnaire developed by the researchers that assessed 3 of the 6 domains recommended in the FSFI standardized questionnaire. The 3 domains evaluated were lubrication, satisfaction, and pain. In the gynecological examination, the clinical signs of vaginal atrophy were investigated, such as discoloration, rugae, petechiae, and friability, subjectively graded from 0 to 2. The 0 graded the mucosa colored, rough, absence or less than 25% of the vaginal walls of petechiae and absent or mild friability (slight bleeding to the touch). The 1 graded pale mucosa, little rough, petechiae 25%‒50% of the vaginal walls, and moderate friability (bleeding evident to the touch). The 2 graded the most intense alterations, pale, smooth mucosa, more than 50% of petechiae on the vaginal walls and intense friability (important bleeding to the touch).

Second visit (45 days after the start of treatment): women were again given orientation about the treatment as well as medication and were scheduled for the third and last visit.

Third visit (90 days after the start of treatment): the women answered the questionnaire described above for the second time, and the second gynecological examination and vaginal biopsy were performed.

The vaginal biopsy was performed with Gaylor forceps in the upper third of the right lateral wall, without the need for local anesthesia. Each biopsy specimen was fixed in 10% formalin. Glass slides were made and stained with hematoxylin-eosin. Histopathological analysis was performed on each sample blindly with respect to temporal correspondence or study group, i.e., the pathologist did not know if the sample was taken before or after the treatment or whether it was from the oxytocin group or the promestriene group.

Epithelial thickness was measured by a pathologist at UNIFESP with experience in lower genital tract pathology, at the thickest section of the sample (considering the proper orientation of the fragment in the paraffin block) and using a conventional light microscope (Nikon Eclipse E200) with 40 ×  magnification. The measurement was performed using a Breslow micrometer, which is used in the microscopic Breslow method, and which has a precision of 0.05 mm, and the results were expressed in mm.

### Statistical analysis

Descriptive statistical analysis was performed for all variables. The results are presented in tables, with absolute frequencies and percentages for the categorical variables. The results of the continuous variables are expressed as mean and standard deviation, median, and interquartile range (75^th^ –25^th^ percentile) and subsequently as the minimum and maximum values (skewness distribution) using the Shapiro-Wilk test.

The following tests were used: Pearson's Chi-Square test or Fisher's exact test for independent group comparisons; Student's *t*-test or Mann-Whitney *U* tests for the comparisons between independent groups and the quantitative variables; Levene's test for comparisons depending on normal distribution and equality of variance; Student's *t*-test or Wilcoxon test for comparisons between before and after within each group (matched or paired data), depending on the existence of normal distribution.

Statistical analyses were performed using the IBM SPSS version 25 (SPSS, Inc., Chicago, IL, USA). The level of statistical significance of the tests for the rejection of the null hypothesis was set at 0.05% or 5%.

## Results

**Figure 1:**
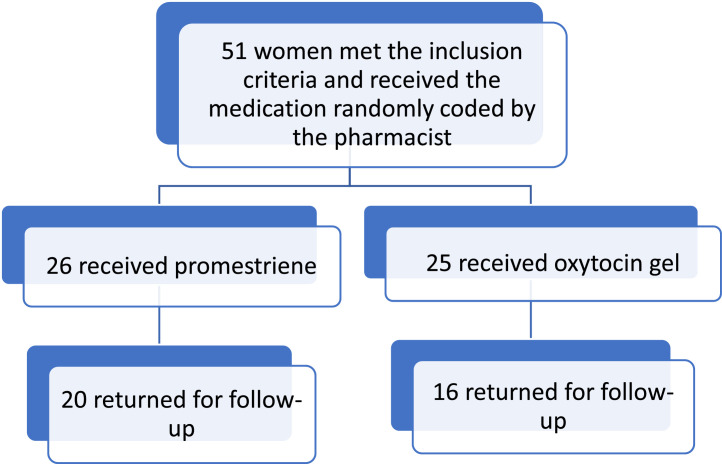
Flowchart of the follow-up of the selected patients.

The study recruited 51 women who met the defined inclusion criteria; 25 patients were allocated to the oxytocin group and 26 to the promestriene group. However, 16 and 20 patients in the oxytocin group and the promestriene group, respectively, completed the study (Fig. 1); 14 patients reported fear of side effects as the reason for abandoning the study, and one patient lost follow-up after moving to another city. That is, the study was developed with a sample of 36 patients.

The median age of the study sample was 54 years and at last menstruation was 47 years; the median number of pregnancies was 3. The majority of the study population defined their marital status as married (74.5%) (Table 1).

**Table 1:** Demographic data and clinical characteristics of the oxytocin and promestriene groups.

	Oxytocin (n = 25)	Promestriene (n = 26)	Total	p-value
Age: Median; IQR (Min–Max)	56; 8 (40–64)	53.5; 8 (45–69)	54; 8 (40–69)	0.515^a^
Number of pregnancies: Median; IQR (Min–Max)	3; 2 (0–8)	3; 2 (0–10)	3; 2 (0–10)	0.049^a^
Age at last menstruation: Median; IQR (Min–Max)	47; 13 (30–55)	48; 6 (30–55)	47; 9 (30–55)	0.491^a^
Marital Status, n (%)				
Single	1 (4)	1 (3.8)	2 (3.9)	0.034^b^
Married	14 (56)	24 (92.3)	38 (74.5)	
In a relationship	3 (12)	0 (0)	3 (5.9)	
Widow	3 (12)	0 (0)	3 (5.9)	
Divorced	4 (16)	1 (3.8)	5 (9.8)	

Note: Data are presented as median and Interquartile Range (IQR). n, absolute frequency; %, Relative frequency (percentage).

a Mann-Whitney test.

b Pearson's Chi-Square test.

When the groups were evaluated separately before and after the treatment, there was a significant improvement in the FSFI domains: lubrication, satisfaction, and pain (p < 0.05) (Table 2).

**Table 2:** Comparison of the FSFI domain scores between the groups before and after treatment with oxytocin and promestriene. Groups evaluated separately (no comparison).

Before	Oxytocin (n = 25)	Promestriene (n = 26)	p-value
	Median; IQR (min‒max)	Median; IQR (min‒max)	
Lubrication	1.5; 2.6 (0–4.5)	1.8; 1.4 (0–3.9)	0.819^a^
Satisfaction	1.6; 3 (0–4)	1.8; 1.6 (0–3.6)	0.894^a^
Pain	1.6; 2 (0–4)	1.2; 1.9 (0–4)	0.985^a^
After	**Oxytocin (n = 16)**	**Promestriene (n = 20)**	**p-value**
	**Median; IQR (min–max)**	**Median; IQR (min–max)**	
Lubrication	3.9; 4.2 (0–4.5)	3.9; 2.9 (0–4.5)	0.422^a^
Satisfaction	3.2; 3.6 (0–4)	3.2; 1.7 (0–4)	0.888^a^
Pain	3; 4 (0–4)	4; 1.5 (0–4)	0.223^a^
Before vs. After	**Oxytocin (n = 16)**	**Promestriene (n = 20)**	
	**p-value**	**p-value**	
Lubrication	0.018^b^	0.005^b^	
Satisfaction	0.036^b^	0.020^b^	
Pain	0.033^b^	0.001^b^	

IQR, Interquartile Range.

a Mann-Whitney test (independent samples).

b Wilcoxon Test (matched samples).

When the groups were compared, there was no difference in FSFI score improvement, i.e., the medications behaved similarly (p > 0.05) (Table 3).

**Table 3:** Comparison of the effects of oxytocin and promestriene on the weighted score of the FSFI domains.

FSFI Domains	Oxytocin (n = 16)	Promestriene (n = 20)	p-value
Lubrication (Mean ± SD)	1.33 ± 1.89	1.46 ± 1.56	0.831^a^
Satisfaction (Mean ± SD)	1.10 ± 1.70	0.78 ± 1.24	0.518^a^
Pain (Mean ± SD)	0.98 ± 1.57	1.54 ± 1.28	0.242^a^

a Student's *t*-test for comparing means of independent samples. Shapiro-Wilk test not significant in both samples for all variables. SD, Standard Deviation.

Although it was observed improvement in clinical examination of the vagina in all patients there was no significance between groups and times.

**Figure 2:**
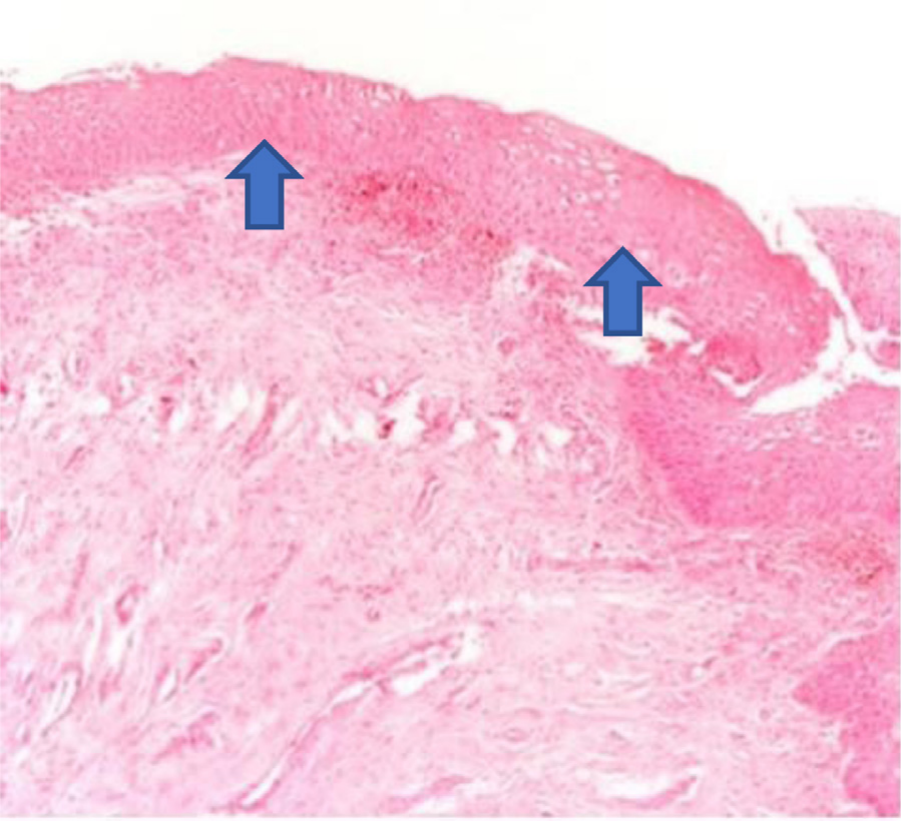
The atrophic epithelium seen in the anatomopathological analysis of vaginal biopsy before oxytocin (arrows). Photomicrographs obtained with an AXIO ZEISS microscope, AxioCam MRc5 system (100 ×  magnification, Hematoxylin–Eosin). Source: Author's archive (2021).

**Figure 3:**
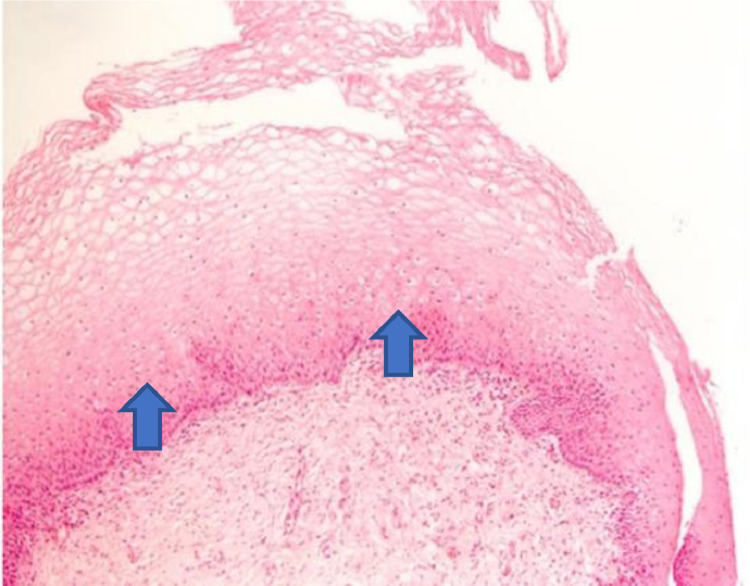
The increase in the thickness of the vaginal epithelium seen in the anatomopathological analysis of vaginal biopsy after oxytocin (arrows). Photomicrographs obtained with an AXIO ZEISS microscope, AxioCam MRc5 system (100 ×  magnification, Hematoxylin-Eosin). Source: Author's archive (2021).

**Figure 4:**
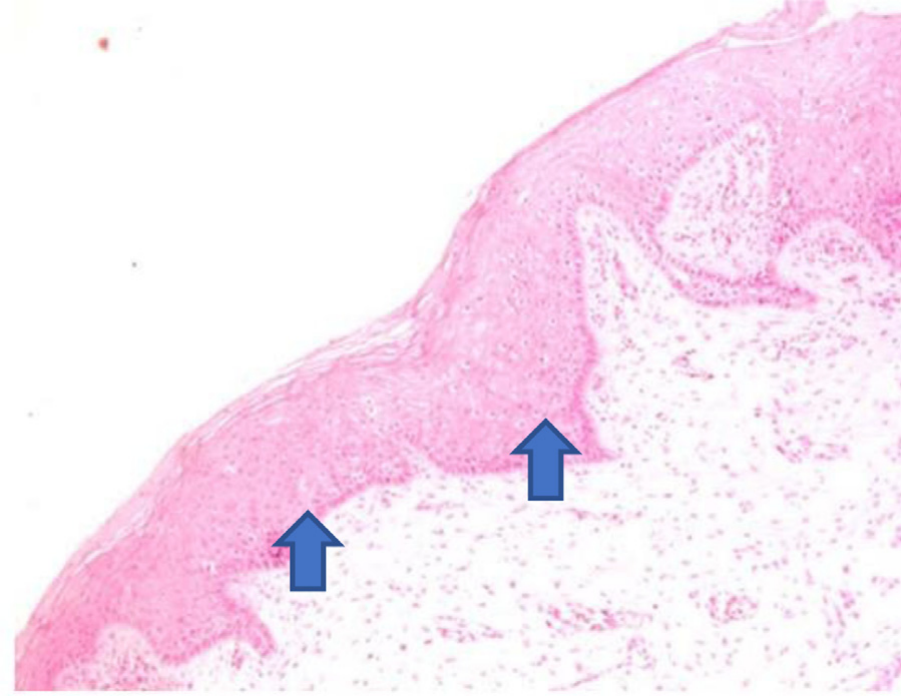
The atrophic epithelium seen in the anatomopathological analysis of vaginal biopsy before promestriene (arrows). Photomicrographs obtained with an AXIO ZEISS microscope, AxioCam MRc5 system (100 ×  magnification, Hematoxylin-Eosin). Source: Author's archive (2021).

**Figure 5:**
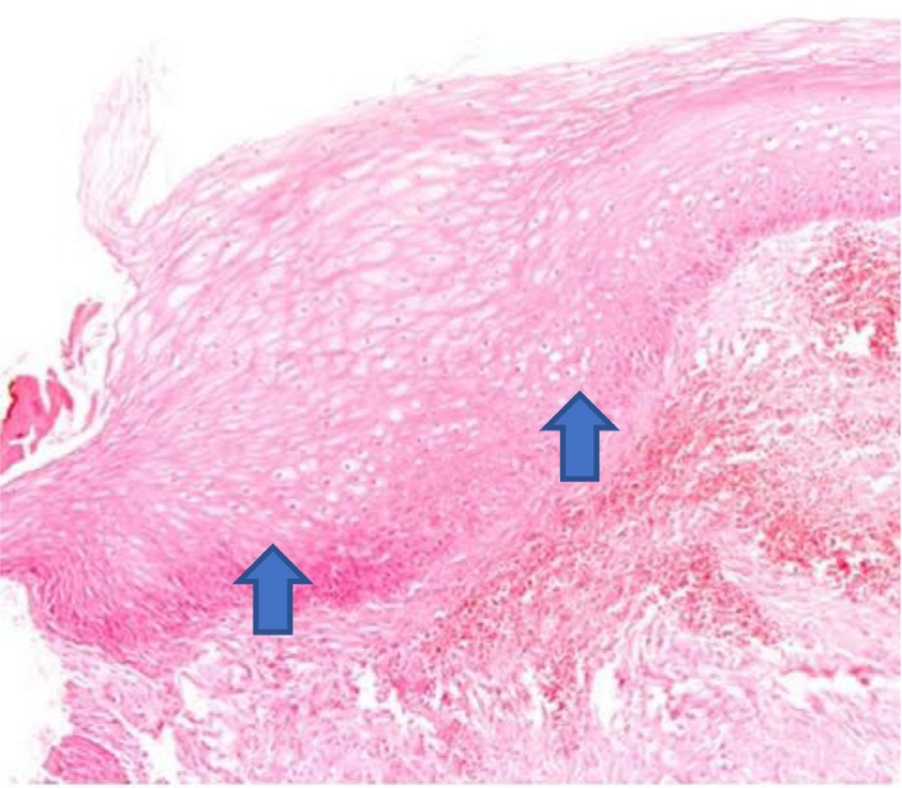
The increase in the thickness of the vaginal epithelium seen in the anatomopathological analysis of vaginal biopsy after promestriene (arrows). Photomicrographs obtained with an AXIO ZEISS microscope, AxioCam MRc5 system (100 ×  magnification, Hematoxylin-Eosin). Source: Author's archive (2021).

The histological analysis of the vaginal wall samples performed before the interventions showed no significant differences in epithelial thickness between the groups (p > 0.05). The comparison between the groups after the treatments showed that both the oxytocin group and the promestriene group had an increase in the vaginal epithelium thickness (p < 0.05). Therefore, both interventions proved to be effective in increasing the thickness of the vaginal epithelium (promestriene p < 0.001 and oxytocin p = 0.017). However, when the medications were compared, the promestriene group showed better efficacy than the oxytocin group (p = 0.036) (Table 4; Figs. 1, 2, 3, Figure 4, Figure 5).

**Table 4:** Comparison of the effect of oxytocin and promestriene on the thickness of the vaginal epithelium.

Thickness of the vaginal epithelium (mm)	Oxytocin mean (SD) Min–Max	Promestriene mean (SD) Min–Max	p-value
Before	(n = 25)	(n = 26)	0.348^a^
	0.21 (0.08)	0.19 (0.07)	
	0.10–0.45	0.05–0.35	
After	(n = 16)	(n = 20)	**0.018**^a^
	0.25 (0.09)	0.32 (0.07)	
	0.10–0.50	0.20–0.45	
Before vs. After	(n = 16)	(n = 20)	**0.036**^a^
Mean (SD)	0.05 (0.08)	0.12 (0.08)	
p-value	0.017^b^	< 0.001^b^	

a Student's *t*-test for comparing means of independent samples.

b Student's *t*-test for comparing means of matched samples. Shapiro-Wilk test not significant in both samples for all variables. SD, Standard Deviation.

## Discussion

In the last decade, oxytocin has attracted the attention of researchers as a promising alternative treatment for GSM.^17^, ^18^, ^19^, ^20^, ^21^^,^^29^ This fact encouraged us to evaluate its effectiveness in relieving symptoms related to postmenopausal urogenital atrophy and to compare it to the well-established effects of promestriene.^16^ So far, there is no research in the literature to date comparing vaginal oxytocin in the treatment of GSM with drugs that have already been proven effective.

A recent systematic review indicated that oxytocin is effective in the treatment of vaginal atrophy, but it emphasized that the use of different doses, the limited number of participants, and the short follow-up period did not allow for a definitive conclusion. Other studies with dose standardization and longer treatment time are required for improving their clinical applicability.^26^

Although research has suggested a positive correlation between the improvement in signs and symptoms with the use of vaginal oxytocin, the results are not consistent in terms of dose and time of use. To date, the conducted studies have been placebo-controlled, the samples have been small, and the duration and dose of the vaginal oxytocin treatment have varied from 100 IU to 600 IU and from 1 to 12 weeks, respectively. In this study, the authors chose an intermediate dose of oxytocin relative to previous studies (300 IU), thus seeking satisfactory results with the minimum possible dose. The authors opted for a 12-week duration, the longest used in previous studies. The vehicle used was a gel, the same as in previous studies, aiming to exclude the potential emollient effect of the cream, which could lead to false results, i.e., to a wrong interpretation.

In this study the authors evaluated the signs and symptoms determined by vulvovaginal atrophy; however, the authors did not assess those related to the urinary tract. According to the demographic data of the study, the groups were homogeneous in terms of age (p > 0.05) and date of last menstruation (p > 0.05).

The authors observed significant improvement in all evaluated domains, i.e., vaginal lubrication, sexual satisfaction, and pain during sexual intercourse, both in the oxytocin group and in the control group (promestriene) (p < 0.05). This confirmed data already reported in the literature by other authors regarding the beneficial effects of oxytocin in the improvement of symptoms related to vulvovaginal atrophy and, as a consequence, in sexual satisfaction.^18^^,^^25^ When the two groups were compared, there was no difference between them (p > 0.05), i.e., both medications were effective.

A pilot study was carried out to initially establish the efficacy of oxytocin in relieving symptoms related to postmenopausal urogenital atrophy and to compare it with the already established effects of promestriene. It is envisaged, in future research, to carry out a study of the non-inferiority of oxytocin in relation to promestriene in women with genitourinary menopause syndrome.

This result is encouraging, considering that promestriene has beneficial effects already established in the treatment of GSM. The present results are also in agreement with the recent results obtained by Abedi et al.,^25^ who evaluated 96 women with vaginal atrophy after the administration of 400 IU of vaginal oxytocin versus placebo for 8 weeks. They noted improvement in the six FSFI domains (desire, arousal, vaginal lubrication, orgasm, sexual satisfaction, and pain or discomfort) with a significant increase in the total sexual function score in the oxytocin group compared with that in the placebo group. The authors concluded that sexual symptoms present in GSM are a valid indication for therapy with oxytocin.^25^

Regarding the clinical examination of the vaginal mucosa, despite the improvement in all parameters evaluated, the results were not statistically significant in both groups (p > 0.05). This may have occurred due to the number of patients allocated to each group and the number of variables analyzed; in fact, since the pioneering pilot study by Jonasson et al.^17^ comparing oxytocin and placebo, a significant improvement in clinical parameters (color, petechiae, and rugae) has been shown in the oxytocin group (p = 0.003).^17^

With regard to the histological evaluation of the vaginal wall, after using the medications, both the oxytocin group and the promestriene group had a significant increase in vaginal epithelium thickness (p < 0.05). Promestriene, however, showed better efficacy than oxytocin (p = 0.036). This result did not surprise us given that the present study compared oxytocin to an estradiol derivative that had already been shown to be effective. The pilot study by Jonasson et al.^17^ showed an improvement of the vaginal epithelium after the use of oxytocin compared with the placebo, but without statistical significance.^17^ However, subsequent studies with larger samples, longer treatment times, and higher doses confirmed with significant results the improvement in symptoms of atrophy and in vaginal epithelium thickness after vaginal oxytocin compared with the placebo.^17^^,^^20^

The authors did not observe adverse effects after using the medications. All patients who abandoned the study reported that the reason was fear of side effects. Vaginal oxytocin has been reported in the literature as a therapeutic option for patients with symptomatic GSM who have contraindications or who are not interested in estrogen therapy. The preliminary results are encouraging, and it is expected that oxytocin will be a further therapeutic option for symptomatic patients with contraindications for hormonal therapy. One of the limitations of this study was the relevant dropout rate of the participants, making the sample studied small. Another limitation was the fact that there are few published studies on the subject.

Thus, based on the preliminary results of this study, the authors will continue this research with a larger number of patients to assess the effects of oxytocin on GSM urinary symptoms and compare them to those of promestriene.

## Conclusions

After 90 days of vaginal application of oxytocin and promestriene, the authors concluded that oxytocin and promestriene were effective, with no significant difference, in improving the FSFI scale scores of lubrications, satisfaction, and pain; improved the alterations in the vaginal mucosa seen on clinical examination, but without statistical significance and were effective in increasing the thickness of the vaginal epithelium, with promestriene being superior.

## Author's contribution

All authors read and approved the final version of the manuscript.

LPA Santos: Responsible for methodology, validation, investigation, data curation, writing, reviewing, editing and visualization.

CE Bonduki: Responsible for formal analysis, writing and supervision.

RCM Dardes: Responsible for writing and supervision.

T. Heink: Responsible for writing and resource acquisition.

MT Patriarca: Responsible for writing, conceptualization, reviewing and editing, and project administration.

## Funding

There is no funding for the work

## Conflicts of interest

The authors declare no conflicts of interest.
